# Author's Responses to Peer Reviews of “The Influence of SARS-CoV-2 Variants on National Case-Fatality Rates: Correlation and Validation Study”

**DOI:** 10.2196/38549

**Published:** 2022-05-24

**Authors:** William A Barletta

**Affiliations:** 1 Department of Physics Massachusetts Institute of Technology Cambridge, MA United States

**Keywords:** SARS-CoV-2, COVID-19, variants of concern, case-fatality rates, virulence, vaccine effectiveness, correlation study

*This is the author’s response to peer-review reports for “The Influence of SARS-CoV-2 Variants on National Case-Fatality Rates: Correlation and Validation Study”*

## Round 1 Review

### Anonymous [1]

#### General Comments

This paper [2] used ecological data to study the correlation between SARS-CoV-2 variants and the fatality rates. It introduced a new indicator to correct for the lagging of the reported death since the initial infection. When applying this indicator to different countries, it demonstrated that the spreading of variants coincided with the surge in death while also acknowledging the potential confounding factors such as vaccination rates. Although the conclusions drawn in this paper showed some inconsistency with other observational/community-based epidemiological studies, the paper also explored the correlation between disease risk factors and the reported death.

Response: Done

The revision makes extensive reference to the “ecological” nature of the data and has revised the analysis and text accordingly (see text in red on the attached PDF).

#### Specific Comments

##### Major Comments

1. The author should provide more characterizations of the proxy case-fatality rate (pCFR). For example, the author should compare the pCFR and the case-fatality rate (CFR) while doing the analysis, such as correlation analysis.

Response: Done

2. The author mentioned “One could equally well average the infection rate over the period from 28 to 14 days,” but no figure was also presented. Comparing different parameters used to construct the pCFR is essential for the reader to evaluate the robustness of the proposed indicator.

Response: Done. The comparison is included.

3. Related to the first point, the author should probably also compare the raw CFR 7-day rolling average and the pCFR 7-day rolling average.

Response: Done

Figure 1 already shows the time variation of the average CFR. Features are very slowly variable and not large in magnitude. Simply dividing the new deaths on day N by the new cases on day N is inappropriate, as those fatalities were from infections contracted several or more days in the past.

4. The death rate is also related to the capacity of the health care system, such as available intensive care unit (ICU) facilities or bed occupancy. Thus, the CFR on a particular day might also depend on the CFR (as an approximation to the ICU occupancy) the day before. While the author reported the absolute pCFR percentage in most of the figures, these results should also be confirmed by replotting the percentages as relative percentages. For example, one could report the daily pCFR as the percentage change to the previous day (or the previous 7-day rolling average).

Response: Done

Actually, the ratio suggested is a differential measurement that is much noisier than the reported time series of the pCFR. A comparison of noise level is easily made by making a Fourier transform of the time series and of the time series of the suggested ratio.

One finds no significant correlation between the pCFR throughout the pandemic versus the national per capita availability of hospital beds. The correlations are shown in Appendix B, Figure B.5.

5. By doing point 4 above, the relative pCFR can be used to compare different included countries that have daily CFRs that are highly variable.

Response: Done

The figure above is a comparison for the United Kingdom. The noise level in the ratio is far too large for this measure to be useful as an absolute measure of increased virulence during waves of diverse variants or to compare differing countries. This figure is reported in Appendix B.

6. The risk factor correlation analysis can be misleading. The author should state very clearly that ecological data were used for the analysis, both in the Introduction and Discussion sections. It has been shown that a population-based correlation provided little insight into understanding the disease pathology. (Portnov B, Dubnov J and Barchana M. On ecological fallacy, assessment errors stemming from misguided variable selection, and the effect of aggregation on the outcome of epidemiological study. J Expo Sci Environ Epidemiol 2007; 17:106-121).

Response: Done. This point has been discussed at length in the revised text. In addition, the “ecological” consideration is the reason for performing a detailed multivariate regression. Some insight into cross-correlations can be gained from a heat map of correlations of the independent variable shown in Appendix C.

7. It is unclear that the definitions of each of the variables (risk factors) are included in the correlation analysis. While I assume it is the same as those cited in the second reference, some of the analysis methodologies seem imprecise. For example, epidemiologists usually model the age as ordinary variables and test for the trend (eg, using ANOVA) but not by using the median age. The author might want to revisit some of the analyses performed.

Response: Done. The three metrics of the age of a country’s population are not imprecise. They are the values given in standard demographic tabular data. These metrics are distinct from the ages of individual patients as analyzed in usual epidemiological data of patient populations. The issues related to the use of any of these age metrics were examined in detail in response to the referee’s point 9.

8. As the author also pointed out, many of these risk factors are correlated with each other. A better way to adjust for these potential confounding effects is by modeling all these risk factors in a regression model.

Response: Done. The author has performed an in-depth correlation and regression analysis of the dependence of pCFR (or even the pandemic average CFR) against a set of 24 independent variables both for the case of the 99 countries of the full study and for the 32 European countries. The heat map of correlations is given in Appendix C. In no case could a model be produced with a P value for the independent variable less than .04. The best model included only coronary heart disease and national health expenditures as the independent variables. The P values for these variables were .04 and .046, respectively. That comment is provided in the text.

9. The author should explain the choice of “shift by 60 days” in Figure 12.

Response: Done. The text now reads “However, shifting the Peruvian distribution 60 days later in time (that is, Day 1 for Peru corresponds to Day 61 for Argentina), increases the correlation of daily new cases in the two countries to 0.86.”

##### Minor Comments

10. The author should consider unifying the color scheme used in the manuscript. For example, some figures are plotted in grayscale, but similar figures can also appear in a colored version.

Response: Done

11. In equation 2, “Total cases on day (N-14) - Total cases on day (N-21),” the “-” between the two phrases can be misleading. The author should consider rewriting the “-” as “to.”

Response: Done

12. The author should also consider replotting the correlation analysis into heat maps. The author did not justify the use of a line plot for plotting each risk factor.

Response: Done. The author agrees that the use of the line chart in Figure 7 is inappropriate. That figure has been replaced with a rank ordered bar chart with separately clustered medical and socioeconomic independent variables.

13. Furthermore, the author should consider clustering the risk factor and plotting a dendrogram with the heat map. Therefore, it will give readers a better idea of the correlation among each risk factor and the correlation among each of the cutoff dates (in Figure 6>) or regions (in Figure 7).

Response: Done. A reduced heat map (Figure 6) emphasizing regional variation is replacing the original Figure 8. A global heat map is given in Appendix C, Figure C.6

### Reviewer BT [3]

#### General Comments

Emerging variants of concern (VOCs) have increased the uncertainty about bringing the pandemic to an end [4]. Countries will not only have to focus on stepping up vaccination efforts but effective surveillance as well to monitor and characterize the more transmissible and deadly variants [5-8]. The most prominent confirmed cases include Alpha, Delta, Beta, Eta, and Kappa [9]. This, in addition to flagging the need for more sustainable measures, raises concerns over their impact on CFRs in different countries.

The authors of the paper “The influence of SARS-CoV-2 variants on national case fatality rates” attempted to investigate the impact of VOCs on (1) pCFRs and (2) the vulnerability of persons living with comorbidities, using open source data of reported daily cases. They found little variations in the association between World Health Organization data-driven factors and the average pCFR and concluded that the increase in the impact of VOCs may be attributed to the fact that those living with comorbidities are more susceptible to infection severity. Other studies that evaluated the impact of new variants found them to be associated with higher rates of hospitalization and death. In the United Kingdom for instance, studies among cohorts infected with the B.1.1.7 variant (VOC-202012/1) compared to those with normal infections found an increased risk of hospitalization [7] and deaths [8,10,11] in the intervention group, using the TaqPath assay. According to expert opinion on some of these results, patients with the Kent or Delta variant (B.1.1.7) were 64% more likely to die [12]. The CFR was higher among men than women and increased with age.

This paper has been structured in compliance with the IMRD approach. The authors capitalized on prior published data and the concept on which the analysis was based [13] to generate new data, which seems logical. The English used is simple enough for the readership but demands improvement.

Even though the paper’s methods and analysis are based on a published concept, the fact that this was done by the same authors and no other authors have been cited making use of the same concept makes the paper’s methods weak. The study rationale has not been well established, thereby making the study objectives and research questions less robust. Besides, not only is data about variants of concern lacking and the interpretation of the results not well articulated, but the conclusion also arrived at is not clear enough in relation to the defined objectives. Kindly refer to the following major and minor comments.

#### Specific Comments

##### Major Comments

1. Kindly refer to the journal guidelines to see how titles are formatted. Well-formatted titles should include the main outcome of interest, the subject matter, and the study design.

Response: Done

2. Your interest is to measure the influence of VOCs, not SARS-CoV-2 variants as reflected in your title. You may want to correct that.

Response: Done

3. Your abstract must include (1) Background, (2) Objective, (3) Methods, (4) Results, and (5) Conclusions. Kindly use this source to see how to structure your paper [14].

Response: Done

4. The phrase I quote “may increase the vulnerability of persons with certain comorbidities” in the Abstract is not an objective. Kindly rephrase together with the first objective that appears too long.

Response: Done

5. You need to include (1) Study Rationale and (2) Specific Objectives in your Introduction as subsections. The “Specific Objectives” subsection should normally be the last part of your Introduction.

Response: Done

6. In your Study Rationale, make efforts to trace other studies that have made use of similar methods in predicting the impact of VOCs. This section needs to at least include some basic data about VOCs (prevalence or impact on hospitalizations and mortality). You may want to make use of this reference [9].

Response: Done. The author has not found similar studies for direct comparison. However, the results of this study are compared with systematic and meta-analyses of clinical studies. As this study does not use characteristics of the structural biology of variants of concern such details would be out of place. However, those details are described in the references cited.

7. Given that this paper is based on VOCs, it would be sensible to include in your Introduction and as part of your background literature evidence of a literature review of the different VOCs (their characteristics and virulence). Readers will be keen to discover the new variants in circulation. The availability of data on VOCs and variants under investigation is key because it flags the need for vaccination, increases uptake, and signals policy makers about the importance of modifying surveillance policies.

Response: Done

8. If you decide to include research questions or hypotheses to be tested in your paper, kindly associate these with your research objectives. This makes it easy for readers to see how you transformed each objective into a question, as well as the hypothesis to be tested.

Response: Done

9. Kindly start your Methods section with the subsection “Study Design” and clearly state your study design. This is particularly important not just for reviewers but for those undertaking systematic reviews.

Response: Done

Studies are often excluded or not simply traced as a result of a lack of a clearly stated research design. Besides, it is the place of the author to inform readers of the study design and not for readers to determine the design that was used. Authors making use of study designs that are new to the journal’s readership always make an effort to cite articles making use of similar designs regarding the subject matter.

10. I suggest structuring your Methods section as follows:

10.1 Study design

10.2 Data sources and setting (including providing a brief description of each country being profiled and the triggers and specific reasons for choosing particular countries to include in your analysis)

10.3 Study variables/outcomes (kindly specify here, the comorbidities you were interested in together with definitions for outcomes like case fatality)

10.4 Data analysis (include equations here and specify any underlying assumptions). Clearly explain how you run the correlations and time series, and report any statistical program that was used.

Response: Done

11. Explain how adjustments for age, sex, ethnicity, type of VOC, seasonality, etc, in the correlations were made. For instance, the impact on the national CFR may be contingent on the type of variant [15]. Comorbidities may exacerbate during winter and make it difficult to attribute increased mortality among those with comorbidities to VOCs [12].

Response: Done. Although the author agrees that the use of sex-disaggregated data would be preferable, a sex-disaggregated and ethnicity-disaggregated data set for COVID-19 has not been reported or is not publicly available in a consistent form for all the countries included in the analysis. The focus on the time series of pCFR and daily infections allows one to observe and, if possible, adjust for seasonal variations. The grouping by region serves as a quasi-proxy for ethnicity data. That explanation is added to the text.

12. In your data analysis, kindly explain how you arrived at using the Pearson product moment correlation. Kindly justify if your data was linear and report the values of normality tests that were performed prior to choosing the approach of analysis.

Response: Done

13. Kindly report how the different linearity assumptions were verified (for linear data).

Response: Done

14. In your data analysis, kindly report how you determined the strength of association between the proxy national CFRs and the different covariates.

Response: Done

15. The Results section seems to be a mix of data analysis, results, and discussion. Kindly move texts relating to the above to their respective subsections. For instance, readers will not expect to see any explanations in the Results section as this should normally appear under discussion, where you normally should explain why results appear the way they are. Additionally, equations relating to data analysis should not appear under results.

Results: Done

16. A look at your study results shows that this paper has 3 objectives I state (1) to assess the fluctuations in the daily proxy national CFRs, (2) to investigate the correlation between average national proxy CFRs and potential cofactors/comorbidities, and (3) to describe the correlation between proxy national CFRs of country pairs by region. You might want to amend your study objectives accordingly.

Response: Done

17. I suggest you organize and report your results by objective (1, 2, and 3) for a better flow.

Response: Done

18. You reported to have made use of the Pearson correlation coefficient but have not reported the coefficients obtained from the correlation anywhere. Kindly clarify.

Response: Done. These correlations among all variables are reported in Figure 5 and in the heat map Figure C.6 in Supplementary Appendix section C.

19. Kindly structure the Discussion section following the journal guidelines. I suggest:

19.1 Summary Findings

19.2 Strength and Limitations

19.3 Interpretation of Results

19.3.1 Fluctuations in the daily proxy national CFRs

19.3.2 Linear correlation of the averaged CFR and potential cofactors

19.3.3 linear correlation between proxy CFRs for country pairs by region

19.4 Implications for Policy and Research

19.5 Conclusion

Response: Done. This section has been restructured.

20. Your need to compare your results with those of other studies in your “Interpretation of Results” in your discussion, by citing other studies on the same subject matter and preferably undertaken in the same countries being profiled. This helps to situate the study within the existing literature. I understand this might be challenging for some objectives. Kindly provide explanations for the results in the event of a lack of suitable studies.

Response: Done where possible

21. Your conclusion needs to state your results within the context of your study objectives and give the significance and implications to future research, surveillance, and policy.

Response: Done

22. Kindly refer to the guidelines for referencing or have a look at published articles in the journal to which this work is submitted. Your references need to follow the AMA citation style. Please refer to the references of this report.

Response: Done

##### Minor Comments

23. The Methods subsection of your Abstract needs to summarize your study design, data sources, and how data was analyzed including any statistical packages.

Response: Done

24. Kindly ensure that the conclusion of your paper is under the subtitle “Conclusion.”

Response: Done

25. Move all abbreviations to the end or as the last section of your paper.

Response: Done

26. Please be aware that you are not allowed to include more than 8 figures in your paper. You may want to merge some and move others to multimedia appendices. I did not find Figure 2 very necessary and you might want to move that.

Response: Done. Figure 2 does illustrate the concept of waves of infection associated with different variants of concern.

27. All figures to be published in the body of your paper must also be uploaded online. Kindly refer to the journal guidelines.

Response: Done

28. I suggest moving Table A to the “Data Sources and Setting” subsection and labeling it as Table 1.

Response: The author considers that including the table in the text would only serve to lengthen the main text while adding little to the description of its contents, which has been added.

Not adopted. This change would enlarge the main text while adding little content.

29. You need to cite more papers including those from the journal to which you submitted.

Response: Done

30. Kindly include a PubMed ID at the end, for each reference (searchable at crossref.org). Kindly refer to the references in this peer-review report.

Response: Done. Included where available.

31. Endeavor to cite the PDF version of articles for all web links if possible.

Response: Done. The DOI of all open access manuscripts cited do include a link to download the PDF of the paper.

### Reviewer CI [16]

#### General Comments

This paper presents the changes in the CFR due to COVID-19 variants in different countries.

#### Specific Comments

##### Major Comments

1. Abstract

1.1. Should include a conclusion section

Response: Done

1.2. Results: A summary of the results in terms of variation in CFR according to the variants needs to be mentioned.

Response: Done

Main Manuscript

2. Objective

2.1. Specify the year for November 1

Response: Done

2.2. Figure 2: What do the different shades indicate? It should be clarified in the footnote. November spelling.

Response: Done

3. Methods of Analysis

3.1. Data sources should be specified for the different countries. The analysis should also mention the methods used for data analysis and presentation in the tables. The data on the infected case load should be used along with the CFR/pCFR.

Response: Done

3.2. pCFR: Full form when used first. The proxy CFR or pCFR should be used consistently in the text.

Response: Done

4. Results

4.1. Figure 7: What was the source of the data for the cofactors in these countries? It should be specified.

Response: Done

4.2. Correlation between regional CFRs

The pairing of the countries should be mentioned in the Methods.

Response: Done

Which statistical test was used for this correlation analysis? This should be mentioned in the Methods

Response: Done

5. Discussions and Conclusions

Discussion and Conclusion should be separated.

Response: Done

### Reviewer CK [17]

I would like to appreciate the author for this study addressing the influence of SARS-CoV-2 variants on national CFRs. The manuscript is concise and well written, and is recommended for possible consideration in its current form. Before publishing the manuscript, I suggest the author presents an Appendix with (a) data with absolute numbers.

Response: Done. Figure 5> and Figure C.6 and Table C.1

(b) Illustration for smoothed values of the pCFR for at least one country (Figures 8-11)

Response: Done. Figures 2, 3, 7, and 8

(c) Discussion on the analytical framework in detail in the Method of Analysis section

Response: Done. The discussion appears in the main text and is extended in the appendix

In conclusion, the subject addressed in this manuscript is worth investigation, and the manuscript is recommended for possible consideration after addressing the above minor concerns.

## Round 2 Review

### Anonymous

This draft has been greatly improved but the author should still consider the following:

1. Rewrite the denominator of equation 11 using the summation sign

Response: Done

2. In the current manuscript, equation 2 appeared before equation 1.

Response: Done. Corrected.

3. There were multiple equation 2s. Equation 1 also appeared twice: in the main text and in the supplementary text.

Response: Done. Corrected.

4. It is better to always mention the year for the date/period that was referenced in the manuscript (eg, “B.1.1.7 (Alpha) and B.1.351 (Beta) strains dated from mid-October and mid-May respectively” and “that could be due to masking by the fraction of Delta cases peaking in Argentina in mid-May” in the Result section).

Response: Done

5. The meaning of the statement “The positive aspect of that limitation is that trends in pCFR can spot burn through cases in unvaccinated of less than vigilant groups” is unclear.

Response: Done. Corrected. The new text reads, “The positive aspect of the sensitivity of the pCFR when case numbers are small is that highly variable trends in pCFR can spot surges of cases in clusters of unvaccinated persons or in less than vigilant groups.”

6. The author mentioned “The red points are due to anomalous entries in the tables of (13)” in the Result section. It would be better to clean the data for the suspected anomalous entries mentioned in the Methods section while plotting the smoothened graph.

Response: Done

Additional smoothing was applied for the April data. All graphs have been updated and improved for clarity.

7. Regression results should be listed in tables that show (at least) effect size and P value.

Response: Done

P values plus the size of effects are now shown for global data in the heat map of Figure 6 and Figure C.5 in the appendix

### Reviewer BT

#### General Comments

I am happy that the authors of the paper titled “SARS-CoV-2 variants of concern: Influences on national case fatality rates” have addressed all concerns raised in the previous round, thereby giving the paper a new and improved outlook. However, these have not been addressed in a manner satisfactory enough. The study title even though modified from “The influence of SARS-CoV-2 variants on national case fatality rates” still needs to comply with the journal guidelines [18]. The study objectives are not consistent across the different sections. Some sections need to be reorganized for a better flow. The English used for reporting warrants improvement. Kindly refer to the below minor comments to improve the paper further.

#### Specific Comments

##### Minor Comments

1. Could you please identify this study as a “Correlation Study” [19]? For instance “The influence of SARS-CoV-2 variants on national case-fatality rates: Correlation and Validation Study”

Response: Done

2. The current text in the Results subsection of the Abstract should be part of the Methods subsection of the Abstract. Kindly move it to the start of your Methods subsection.

Respond: Done

Could you please summarize your findings into say 5 to 10 lines in the Results section of your Abstract? One will expect to see some figures reported from the main results in this subsection. You may want to ensure that your word count for the Abstract is not above 450 by decreasing the word count in your Methods and Conclusions subsections.

Response: Done

3. The discoverability of your paper can be improved by including SARS-CoV-2, COVID-19, and 2019-nCoV in your keywords. Kindly modify “Country correlation” to “Correlation study.”

Response: Done

4. The Objectives section of your Introduction seems to include the study background information; otherwise, I do not understand why it should be that lengthy. Kindly move the subtitle “Objectives” (better phrased as “Specific Objectives”) to the end of your Introduction and state your specific objectives. The Objectives subsection should not be more than a paragraph. All other text should either be part of your study background literature or rationale. The Specific Objectives subsection should be formatted as follows:

Specific Objectives

The principal objectives of this study are to (1) establish a valid proxy national CFR and assess its daily fluctuations, (2) investigate the correlation between average national proxy CFRs and potential cofactors/comorbidities on a global and regional basis, and (3) describe the correlation between proxy national CFRs of country pairs by region.

Response: Done

Please do not include any other text before the Methods section. Additionally, kindly ensure that the above specific objectives and those in your Abstract are the same for consistency.

Response: Done

5. The use of the word “reference” in most of your statements (eg, “To evaluate any changes in the susceptibility to co-factors, one can follow the method introduced in reference”) may not be appropriate. I suggest you state author names instead of using “reference” when referring to a particular research work. Kindly rephrase these all through the body of the manuscript.

Response: Done

6. For standard reporting and to be in line with the journal guidelines, I suggest replacing the title “Method of Analysis” with “Methods.” It will be good to identify this study as a “Correlation and Validation” study under your “Study Design” subsection. This should be a single statement or at most 5 lines if you need to explain why you used the design and make reference to other papers.

Response: Done

7. Regarding your analysis approach in the study methods, it will be good to provide a few lines on how each of the assumptions for running a Pearson product moment correlation was satisfied [20].

Response: Done

This is described in steps B through D of the methodology.

8. Kindly change the title “Discussion and Conclusion” to “Discussion.” I still suggest you structure your Discussion in line with the journal guidelines [21]. You may want to refer to papers published in JMIR to help you with how to structure the Discussion section. Based on journal guidelines, well organized and standard Discussion sections will bring out the subtitles (not as paragraphs) “Summary of Findings,” Study Limitations,” “Comparison With Prior Studies,” and the “Conclusion.” Even in a situation where you do not have enough papers to cite under “Comparison With Prior Studies,” the subsection will still include your reasons and explanations of why results appear the way they do.

Response: Done

9. I guess your current Conclusion that appears quite lengthy includes materials for the Discussion section. Kindly size down and move a majority of the material to the Discussion section (specifically to the “Comparison With Prior Studies” subsection).

Response: Done

10. I note that the “Summary of Findings” in the Discussion should be a carbon print in terms of length and text of the “Results” subsection in the Abstract. For coherence and consistency, the more you can make these the same, the better. The same should be the case with the “Objectives” subsection in the Abstract and the “Specific Objectives” subsection at the end of your Introduction.

Response: Done

11. Kindly define a study aim in one sentence based on your 3 specific objectives and start your Conclusion with this study aim. This reminds readers of what you set out to do and helps them marry it with what you found. This should be followed by the main findings in just a few lines, lessons learned, what the findings mean for public health, and future research.

Response: Done

12. Just like the “Summary of findings,” it is common practice not to expect the Conclusion of a paper to be lengthy since all explanations relating to the results should be part of your “Comparison With Prior Studies” subsection in the Discussion.

Response: Done

13. As per the journal guidelines, kindly move your Abbreviations subsection to after the references.

Response: Done

14. Ensure you follow the journal guidelines to report your P values.

Response: Done

## Abbreviations

CFR: variant of concern
ICU: intensive care unit
pCFR: proxy case-fatality rate
VOC: variants of concern
